# Analysis of a beta-lactam allergy assessment protocol challenging diverse reported allergies managed by an antimicrobial stewardship program

**DOI:** 10.1017/ash.2023.432

**Published:** 2023-09-08

**Authors:** Max W. Jacobs, Derek N. Bremmer, Nathan R. Shively, Matthew A. Moffa, Tamara L. Trienski, Dustin R. Carr, Carley A. Buchanan, Thomas L. Walsh

**Affiliations:** 1 Medicine Institute, Allegheny Health Network, Pittsburgh, PA, USA; 2 Department of Pharmacy, Allegheny Health Network, Pittsburgh, PA, USA; 3 Division of Infectious Diseases, Allegheny Health Network, Pittsburgh, PA, USA

**Keywords:** allergy, allergy delabeling, antibiotic, antimicrobial stewardship program, beta-lactam, penicillin

## Abstract

**Objective:**

To assess the safety and efficacy of a novel beta-lactam allergy assessment algorithm managed by an antimicrobial stewardship program (ASP) team.

**Design:**

Retrospective analysis.

**Setting:**

One quaternary referral teaching hospital and one tertiary care teaching hospital in a large western Pennsylvania health network.

**Patients or participants:**

Patients who received a beta-lactam challenge dose under the beta-lactam allergy assessment algorithm.

**Interventions:**

A beta-lactam allergy assessment protocol was designed and implemented by an ASP team. The protocol risk stratified patients’ reported allergies to identify patients appropriate for a challenge with a beta-lactam antibiotic. This retrospective analysis assessed the safety and efficacy of this protocol among patients receiving a challenge dose from November 2017 to July 2021.

**Results:**

Over a 45-month period, 119 total patients with either penicillin or cephalosporin allergies entered the protocol. Following a challenge dose, 106 (89.1%) patients were treated with a beta-lactam. Eleven patients had adverse reactions to a challenge dose, one of which required escalation of care to the intensive care unit. Of the patients with an unknown or low-risk reported allergy, 7/66 (10.6%) had an observed adverse reaction compared to 3/42 (7.1%) who had an observed reaction with a reported high-risk or anaphylactic allergy.

**Conclusions:**

Our implemented protocol was safe and effective, with over 90% of patients tolerating the challenge without incident and many going on to receive indicated beta-lactam therapy. This protocol may serve as a framework for other inpatient ASP teams to implement a low-barrier allergy assessment led by ASP teams.

Beta-lactam allergy assessments are important initiatives for healthcare institutions to promote, and there are a large diversity of delabeling protocols.^1^ Penicillin allergies are the most commonly reported antibiotic allergy in the United States with 10–15% of all hospitalized patients reporting an adverse reaction to penicillin.^2^ This is problematic because patients with listed penicillin allergies have worse clinical outcomes, including increased incidence of hospital admissions, infections from drug-resistant organisms, intensive care unit (ICU) admission, mechanical ventilation, in-hospital mortality, as well as longer hospital lengths of stay which is attributed to suboptimal antibiotic therapy.^3–5^ Furthermore, many listed allergies are not accurate and/or clinically relevant, and over 80% of patients with true IgE-mediated penicillin allergy will lose this response after 10 years.^6^ Despite potential concerns for cephalosporin cross-reactivity, patients with a penicillin allergy may have as low as a 0.12% chance of having a reaction to a third-generation cephalosporin.^7–9^ Thus, the vast majority of patients with a listed beta-lactam allergy can be safely treated with beta-lactam agents.

The American Academy of Allergy, Asthma, and Immunology, the American College of Allergy, Asthma, and Immunology, and the Infectious Disease Society of America all recommend implementation of penicillin delabeling protocols.^10,11^ Previously described protocols involve allergen skin testing, beta-lactam test dosing, and delabeling based on history alone.^12^ Nonallergic reactions such as headache, family history of reactions, or diarrhea can generally be removed without the need for testing. Skin testing, which is the gold standard, is recommended for those with severe IgE-mediated reactions such as anaphylaxis. The prospect of widespread allergen skin testing is limited by available personnel as well as time and financial constraints of test administation^13,14^. Several studies have shown penicillin challenge doses are safe and appropriate for patients with benign rashes and mild symptoms, but there is not robust data on challenging patients with more severe reported reactions.^15–17^ Determining who qualifies for challenge doses is often a multidisciplinary effort that can include immunology and allergy specialists, clinical pharmacists, infectious disease (ID) specialists, and antimicrobial stewardship teams.^18,19^

We describe the real-world impact of a beta-lactam allergy assessment algorithm which was developed, disseminated, and implemented in two hospitals in a large western Pennsylvania health network.

## Methods

This was a retrospective analysis of a beta-lactam allergy assessment that was used in two large hospitals in western Pennsylvania between November 2017 and July 2021. The assessment was driven by the antimicrobial stewardship program (ASP) team. Patient eligibility was determined by ASP review and discussion with the primary care team or by ID consultation. Data were generated by a report that identified any patient who utilized the order set for a graded challenge. We sought to report the safety and efficacy of this protocol by collecting data on patients undergoing a graded challenge, agent used for the graded challenge, and reaction type and severity to graded challenge. This analysis was deemed quality assessment and quality improvement by our local institutional review board.

### Facility details

Allegheny General Hospital is a 576-bed, urban teaching hospital in Pittsburgh, Pennsylvania. It is Allegheny Health Network’s flagship, quaternary care center and sees over 24,000 annual admissions. West Penn Hospital is a 317-bed, tertiary care teaching hospital also in Pittsburgh, Pennsylvania, and also a part of Allegheny Health Network. The ASP team is comprised of four full-time clinical pharmacist specialists and three ID physicians. There are three ASP clinical pharmacists at Allegheny General Hospital, one ASP clinical pharmacist at West Penn Hospital, and the three ID physicians split their clinical and ASP dedicated time between both hospitals. Both hospitals have on-site ID consult teams who are in close communication with the ASP team. Each hospital has available allergy and immunology specialists who originally approved the protocol but were not involved with individual patient recommendations of the protocol.

### Beta-lactam allergy assessment protocol

**Figure 1.**
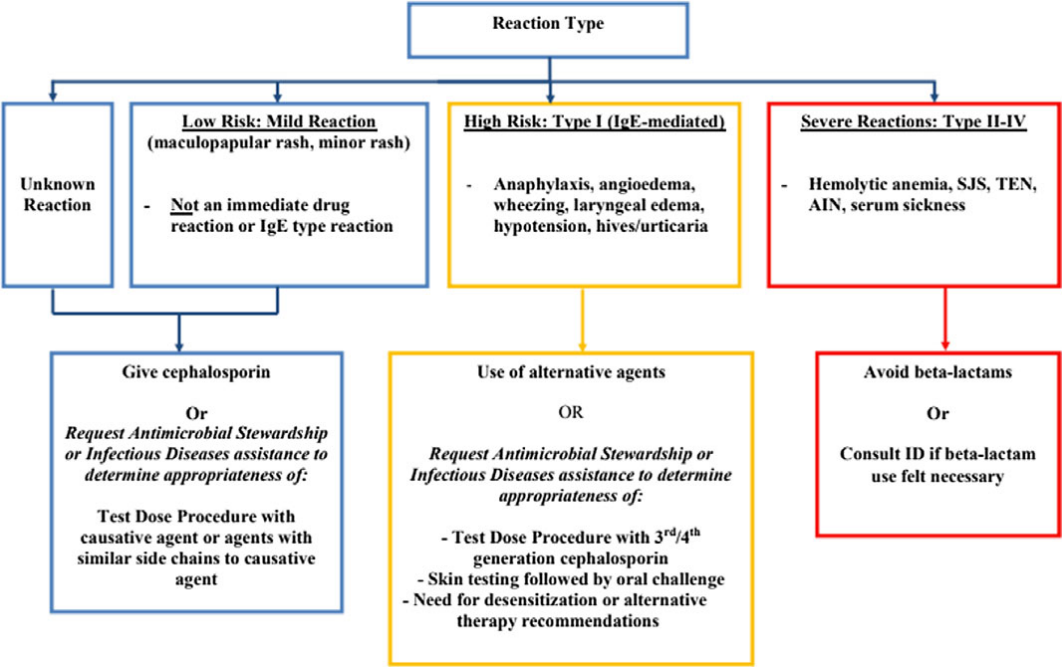
Beta-lactam allergy assessment algorithm.

At the implementation of the protocol, several lectures were given to educate practitioners from different departments on the initiative. A patient was eligible for the protocol if they had a listed beta-lactam allergy, were not pregnant, and over 18 years old. A detailed history was taken of the patient’s beta-lactam reaction. A low-risk mild reaction was defined as a maculopapular or mild rash or an unknown reaction. High-risk, type I IgE-mediated reaction included anaphylaxis, angioedema, wheezing, laryngeal edema, hypotension, hives, or urticaria. Severe, types II–IV, reactions included serum sickness, Steven–Johnson syndrome (SJS), toxic epidermal necrolysis, acute interstitial nephritis, or hemolytic anemia (Figure 1). Although it was not necessary for a patient to have an active bacterial infection to be included in our protocol, the main route that patients were identified was due to receipt of a non-beta lactam agent due to reported beta-lactam allergy via routine ASP team review of patients on antibiotics or via formal ID consultation.

Patients with a reported severe reaction were not offered a graded challenge. Patients with either low-risk or high-risk reactions were offered a graded challenge which was built into an order set in the electronic medical record (EMR). A challenge dose of an intravenous (IV) or oral antibiotic was 10% of the ideal treatment dose. Patients with reported low-risk reactions received a challenge dose which could have been the offending agent or an agent with a similar side chain. Patients with reported high-risk reactions received an antibiotic preferably with a different R1 group. Bedside registered nurses (RNs), without direct physician oversight, administered the challenge doses and monitored the patient for adverse reactions. This was performed on the unit to which the patient was admitted without transfer to a higher level of care prior to initiating the graded challenge. For high-risk reactions, there was also an option to consult ID or ASP for the consideration of skin testing and/or a desensitization protocol.

**Figure 2.**
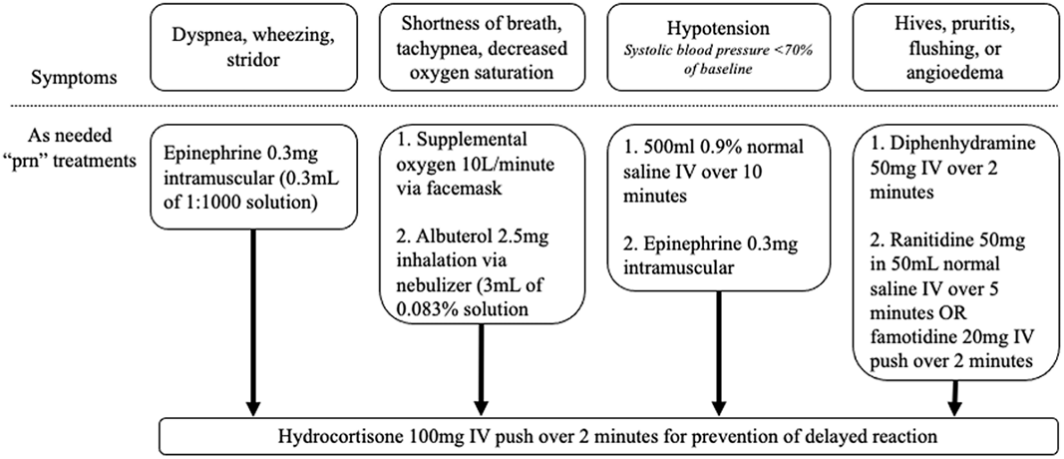
Treatments available, as needed, for potential reactions during the beta-lactam allergy assessment protocol.

The protocol is started by recording baseline vital signs and then administering the challenge dose. Vital signs were recorded 30 and 60 minutes afterward. If there was no adverse reaction, a full treatment dose was administered and vital signs were again measured at 30 and 60 minutes afterward. Throughout the protocol, each patient had an “as needed” order set with accompanying medications for the management of possible adverse reactions (Figure 2). The date, antibiotic used, and tolerance or intolerance to the graded challenge were documented in the EMR. If a skin test was going to be used, beta-blockers and angiotensin-converting enzyme inhibitors were held the day of the procedure. The listed allergy was updated to included the date, details, and results of the challenge. The allergy was not deleted to prevent relisting during a subsequent encounter between the patient and a new clinician.

## Results

Over the 45-month study period, a total of 119 patients entered the protocol, for an average of 2.6 patients per month. Penicillin allergy was reported in 101 patients and 18 patients reported a cephalosporin allergy (Table 1). The most common reported reactions were rash (n = 43) and anaphylaxis (n = 42). The most commonly used antibiotic for a graded challenge was amoxicillin (34.5% of the challenge doses), followed by cefdinir (26.9%), ceftriaxone (8.4%), and cefepime (8.4%). Over half of the challenge doses were cephalosporins (55.5%).

**Table 1.** Patients treated with the beta-lactam allergy assessment algorithm

Variable	n (total = 119)	%
Setting		
General wards/non-ICU	101	84.9%
ICU	18	15.1%
Reported allergic agent		
Penicillin/amoxicillin	101	84.9%
Cephalosporin	18	15.1%
Reported reaction		
Unknown	23	19.3%
Rash	43	36.1%
Anaphylaxis	42	35.3%
Other^a^	11	9.2%
Challenged with		
Amoxicillin	41	34.5%
Ampicillin	8	6.7%
Cephalosporin^b^	66	55.5%
Other^c^	4	3.4%
Observed immediate reaction to test dose	4	3.4%
Beta-lactam prescribed following test dose	106	89.1%
Observed delayed reaction	7	5.9%
Long-term treatment with beta-lactam, > 14 days	52	43.7%

a Non-anaphylactic swelling (6), dyspnea (1), nausea with vomiting (1), diarrhea (1), fever (1), and pruritus (1).

b Cefdinir (32), ceftriaxone (10), cefepime (10), cefazolin (7), cephalexin (6), and cefuroxime (1).

c Penicillin (2), piperacillin-tazobactam (1), and skin testing only (1).

Overall, 11 patients had an observed reaction (Table 2); four patients (3.4%) had an immediate reaction within 24 hours of the first challenge dose and 7 patients (5.9%) had a delayed reaction. Of the five patients who had immediate reactions, there was one case of a rash, two cases of flushing, and one case of pruritus without rash (Table 2). Of the seven patients who developed delayed reactions, five of which developed a rash, one had pruritus, and one had facial swelling and shortness of breath. None of the rashes had symptoms concerning for SJS or other severe reactions. Descriptions of the rashes included “mild,” “low-severity,” “maculopapular,” and “nodular.” One of the patients who had delayed reactions causing a rash required escalation of care to the ICU. This patient had initially tolerated a challenge with ampicillin and was discharged. Six days after the initial challenge, the patient presented to the hospital with rash and hypotension requiring ICU care. They were treated with diphenhydramine and epinephrine and made a full recovery. This rash was thought to be a delayed reaction from ampicillin, but drug reaction with eosinophilia and systemic symptoms (DRESS) from concurrent vancomycin was also considered. This patient had tolerated vancomycin on previous admissions without any reported adverse reactions. Of the 11 patients who had observed reactions following the challenge, 5 received beta-lactam antibiotics after the observed reaction (Table 2).

**Table 2.** Documented reactions to test doses: the 11 patients who had reported reactions during the beta-lactam allergy assessment protocol

Reported allergy	Reported reaction	Challenged with	Immediate reaction	Delayed reaction^a^	Treatment	Escalation of care?	Beta-lactam prescribed after?
Ampicillin	Anaphylaxis	Cefdinir	Rash	None	Diphenhydramine	No	No
Penicillin	Rash	Amoxicillin	Pruritus	None	Diphenhydramine hydrocortisone	No	No
Penicillin	Unknown	Ceftriaxone	Flushing	None	Diphenhydramine hydrocortisone	No	No
Ceftriaxone	Rash	Ceftriaxone	Flushing	None	Diphenhydramine famotidine	No	Ertapenem
Cefazolin	Rash	Cefepime	None	rash	Diphenhydramine	No	Piperacillin /tazobactam
Penicillin	Rash	Cephalexin	None	Rash, pruritus	Diphenhydramine	No	No
Penicillin	Anaphylaxis	Cefdinir	None	Rash	None	No	Cefepime
Penicillin	Anaphylaxis	Cefdinir	None	Pruritus	Diphenhydramine	No	Cefdinir
Penicillin	Rash	Cefdinir	None	Rash	None	No	Cefepime
Penicillin	Rash	Ampicillin	None	Rash	Diphenhydramine and epinephrine	Yes—ICU	No
Penicillin	Difficulty breathing	Cefazolin	None	Swelling of cheeks and dyspnea	Diphenhydramine and famotidine	No	No

a Delayed reaction is a reaction occurring >24 hours after challenge dose administration.

**Figure 3.**
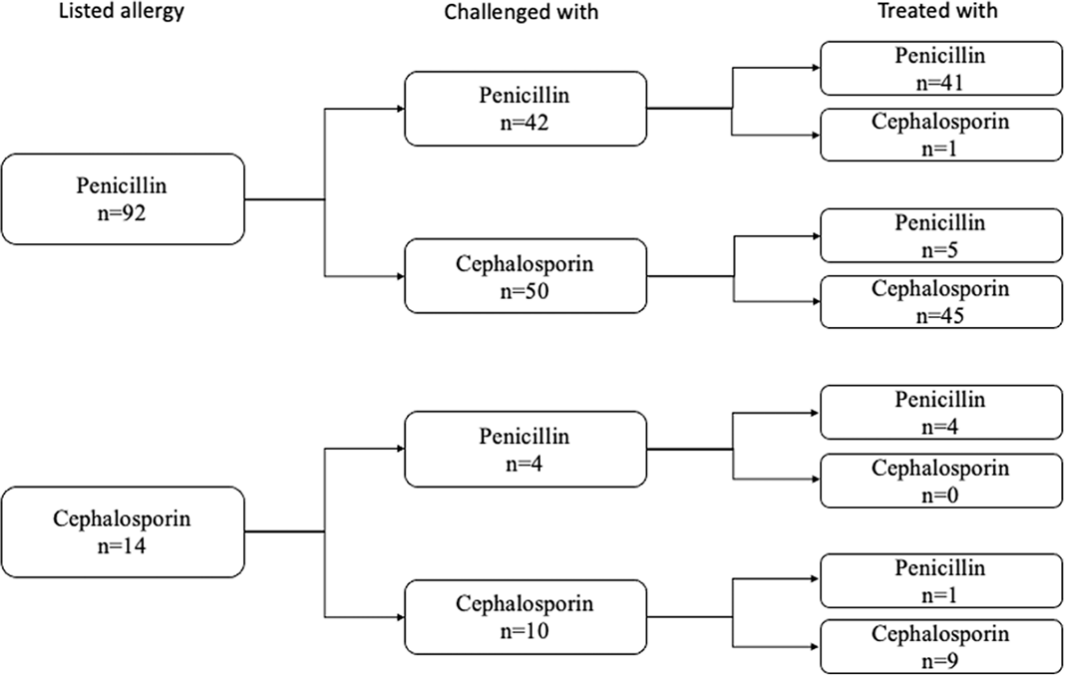
The portion of patients with observed adverse reactions after a beta-lactam challenge stratified by the severity of their reported allergy.

Seven out of sixty-six (10.6%) patients with unknown or low-risk reported allergies had an observed adverse reaction after the allergy assessment protocol. Three out of forty-two (7.1%) patients with anaphylaxis or high-risk reported allergies had an observed adverse reaction. One out of eleven (9.1%) patients with other reported allergies had an observed reaction (Figure 3).

**Figure 4.**
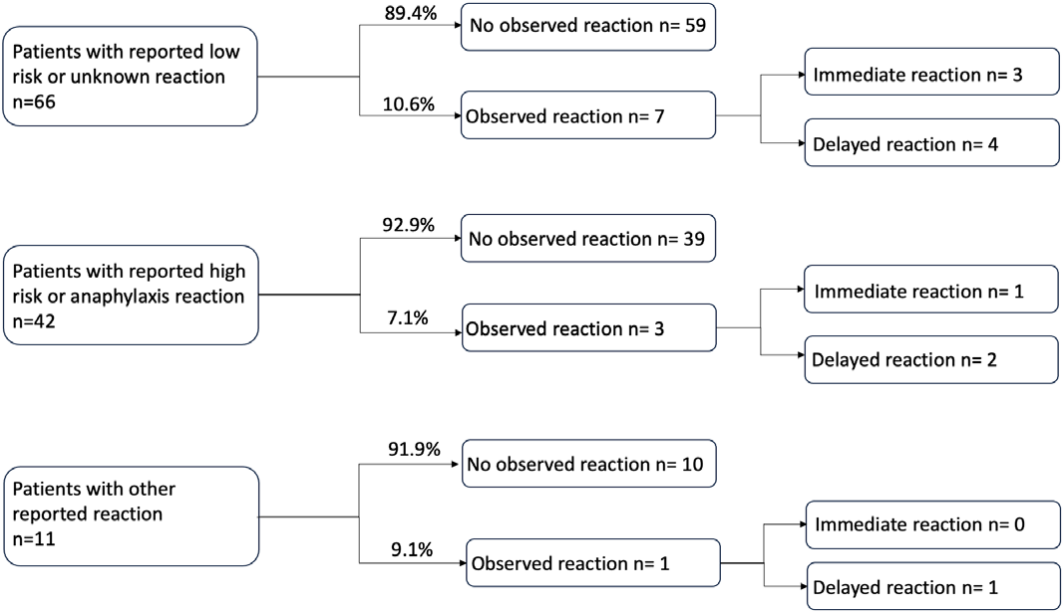
The 106 patients who were treated with beta-lactams after the successful challenge, subdivided by types of reported antibiotic allergy, the type of antibiotic with which they were challenged, and the type of antibiotic with which they were ultimately treated.

Following a challenge dose, 106 (89.1%) patients were treated with a beta-lactam antibiotic and 52 (43.7%) were treated with long-term courses (> 14 days of treatment). The remainder had a change of clinical course and no longer required a beta-lactam antibiotic. The majority (50/99, 51%) of the prescribed antibiotic courses were from the same subtype of antibiotic that was listed as the patient’s allergy (Figure 4). Two patients received skin testing. One of those patients received follow-up graded beta-lactam challenge doses. Both were ultimately prescribed beta-lactams without reported reactions.

ID was consulted on 108/119 patients (90.7%) of the patients. There were not any adverse reactions in the 11 patients who underwent a challenge without an ID consult. This included six patients with reported unknown or low-risk reactions and five patients with reported high-risk reactions.

## Discussion

After the implementation of a beta-lactam allergy assessment algorithm, we found the graded challenges were safe and effective, with over 90% of patients tolerating the challenge without incident and many going on to receive the indicated first-line beta-lactam therapy. The most significant findings of our allergy assessment protocol include its overall safety, effectiveness in delabeling high-risk allergies, efficacy without the need for skin testing or allergy specialists, and integration with the existing ASP. The small portion of patients who had reactions had mild symptoms. There was one patient who required escalation of care with ICU care, but the relation to beta-lactam allergy assessment is unclear. It is important to recognize that even patients with reported anaphylaxis as their allergies did not experience a higher rate of adverse effects or any advanced monitoring such as physician presence at bedside or transfer to the ICU for the challenge. Although side chains are molecularly the most appropriate categorization when considering a cross-reaction between beta-lactams, there is often unsubstantiated concern about penicillin and cephalosporin class cross-reactions. An analysis of subtypes, penicillins and cephalosporins, was produced in order to reinforce that there is low cross-reactivity.

There are several previously described penicillin allergy challenges with well-documented success and safety profiles. Commonly, they are performed with skin testing under the direct supervision of trained allergist physicians^20,21^. Although effective, these protocols may be cost-prohibitive and there is concern that smaller health systems may not have readily accessible allergists.^22^ Other protocols are designed without skin testing and are carried out by nonallergists but exclude reported high-risk allergies.^16,23,24^ Our protocol was designed based on a previously described inpatient allergy assessment that also minimizes skin allergy testing by Blumenthal et al., although key differences exist.^25^ The Blumenthal protocol challenged all low-risk reactions with a penicillin, the first-generation cephalosporin or the second-generation cephalosporin. Our protocol also allowed patients to be challenged directly with the reported offending agent, even if it was not part of one of those classes. The Blumenthal protocol also offered alternative antibiotics if challenging was not pursued, aztreonam for low-risk reactions and carbapenem for high-risk reactions. These options were not available in our protocol. Unknown reactions followed the low-risk workflow in our protocol instead of the high-risk protocol. Finally, our study population had a larger proportion of patients with a reported high-risk or anaphylactic reaction.

Our protocol design has three benefits: minimal use of skin testing, challenging reported anaphylaxis reactions, and bedside testing without direct specialist involvement. Although skin testing was technically part of our protocol, it was utilized in < 2% of patients and could likely be avoided altogether in further iterations. More skin testing was not pursued due to overall safety of high-risk patients receiving a challenge dose and limited number of patients requiring treatment with an agent to which they had a high-risk reaction. The true overall prevalence of beta-lactam allergy is unknown. But within the population of people with reported penicillin allergy, our protocol had rates of adverse reactions similar to other graded challenges protocols.^16^ Graded challenge protocols tend to have higher rates of reaction than protocols that rely on skin allergy testing.^26,27^ One explanation is that during a graded challenge, a reaction may be incorrectly associated with the challenge medication. Often times graded challenges are given in environments, such as our protocol, where several other plausible antigens could be contributing. Patients with reported high-risk reactions, including anaphylaxis, were included in our protocol. These patients did not have higher rates of reactions to challenge dosing, suggesting that allergy assessment protocols may be safely implemented to a wider range of patients than previously included. This protocol was followed in the inpatient setting without anticipatory escalation of care (i.e. preemptive transfer to step down or intensive care unit) and without a physician being present. To this point, over 80% of the patients had their challenge doses administered while not in an ICU. Overall, this design creates a low-barrier protocol for both patients and clinicians; more patients are included without superfluous use of hospital resources.

In addition to a unique design, the implementation by an ASP makes this protocol widely applicable as a pragmatic solution. In 2017, the Joint Commission mandated that all hospitals in the United States establish an ASP, a decision that has since been supported and required by Centers for Medicare and Medicaid.^28,29^ With these teams already in place, ASP-led beta-lactam allergy challenge protocols may be an ideal solution for many US healthcare centers. Pharmacists, especially clinical pharmacy specialists, are uniquely positioned to help implement and guide such a program, given their expertise in medication management. We are hopeful that our described results provide confidence for this protocol to be duplicated elsewhere.

This study had several limitations. First, it was a retrospective, observational study to evaluate a protocol after implementation. Patients were likely selected based on clinical convenience of either the treating clinician or the ASP team. As patients for inclusion in this study were identified via graded challenge order set utilization, we cannot report how many patients were considered for graded challenges verses how many received a challenge. This methodology also likely led to an underestimation of the benefit of the protocol, as we are not able to quantify how many patients were able to have beta-lactam allergies removed by history alone, negating the need for a graded challenge. The small sample size is also a limitation of this study. Finally, there was no standardized way to quantify the severity of reactions to the challenge drug. Reactions were documented based on a predefined set of questions, but there was still room for subjectivity from the bedside nurse and treatment teams.

Our findings add to the body of evidence that beta-lactam allergies are likely overreported and can safely be challenged. This protocol can act as a framework for other inpatient facilities to implement a low-barrier allergy assessment led by ASP teams.
